# Quantum cascade laser: 30 years of discoveries

**DOI:** 10.1515/nanoph-2025-0482

**Published:** 2025-10-21

**Authors:** Miriam S. Vitiello, Jerome Faist, Benjamin S. Williams, Paolo De Natale

**Affiliations:** 9327 NEST, CNR-Istituto Nanoscienze and Scuola Normale Superiore, Piazza San Silvestro 12, 56127, Pisa, Italy; Institute for Quantum Electronics, ETH Zürich, Zürich, Switzerland; Department of Electrical and Computer Engineering, University of California at Los Angeles, Los Angeles, CA, 90095, USA; CNR-INO – Istituto Nazionale di Ottica, Largo E. Fermi, 6, I-50125, Firenze, Italy

Thirty years after their discovery, quantum cascade lasers (QCLs) remain highly relevant due to their unique capabilities and continued advancement in mid- and far-infrared photonics.

The first quantum cascade laser was demonstrated in Bell Laboratories in 1994.

This device featured characteristics that distinguished it from all the other semiconductor lasers. It was unipolar, based on the transport of a single type of carriers that did not involve transitions between electrons and holes across a bandgap. It shared that characteristic with the cyclotron resonance laser, a device first proposed by B. Lax in 1960 and then realized using extremely pure p-doped germanium in a cross electric and magnetic field. Unfortunately, the p-germanium laser required cryogenic cooling and a relatively large pumping power.

In contrast to that device, the quantum cascade laser [1] gain region was based on transitions between electronic subbands defined by quantum confinement in a semiconductor heterostructure. It shared the gain mechanism with the celebrated proposal of Kasarinov and Suris [2] who investigated intersubband gain in a strongly biased superlattice. As a result, the quantum cascade laser offered the promise of unparalleled flexibility in designing devices over a wide frequency range covering the terahertz and mid-infrared regions.

The papers presented in the issue “*Quantum Cascade laser: 30 years of discoveries*” are contributions selected from the IQCLSW, the biannual QCL dedicated conference summoning since 2004 the QCL community, held at Ischia in 2024, and a symposium, IQCLS, held in Zurich, more focused to the 30^th^ anniversary of QCLs, presenting the advances in the field of quantum cascade laser research 30 years after its first demonstration.

These reports highlight the vitality of the field and illustrate the fact that this device delivered on most of the promises made at its birth.

Very quickly after its first demonstration, the QCL demonstrated operation at room temperature, first in pulsed operation and then in continuous wave. It remained the only semiconductor laser operating under ambient conditions in the mid-infrared range for many years, recently rejoined by the interband cascade laser. The development of such mid-infrared sources continues with recent works on small dissipation ring interband QCL [3], the integration of these devices with other mid-infrared optical elements using low-loss [4] or plasmonic waveguides [5] for integrated sensing applications. Besides their use for sensing, the capability of the QCL to be modulated at very high frequencies as well as its large optical power makes it a very capable device for telecommunication and lidar applications [6].

Another important milestone was the extension of the operation wavelength from the mid-IR to the terahertz (THz) frequency range for both detectors and lasers, as reviewed in Ref. [7] by *Q. Hu*. The maximum operation temperature of these devices is still continuously improving, and now THz QCLs are specifically optimized for operation on a Peltier element, such as reported in Ref. [8]. A very interesting aspect of ongoing research on such devices is their integration with other THz optical elements, such as photoconductive antennas [9]. The highly confined nature of the waveguide also enables the test of very advanced photonic structures, such as bound states in the continuum, which provide optical modes with an extremely high quality factor [10].

The very fast gain recovery time of the gain medium, as well as the capability of designing the dynamics of the active region using quantum engineering, makes the quantum cascade laser a very interesting device for mode-locking application. Careful modeling of the electronic distribution and its dynamical behavior in the device are reviewed and theoretically described in Ref. [11]. While the fast recovery time hampers the generation of high intensity pulses, the very strong non-linearity it introduces in the gain medium, which one can see as a very effective four-wave mixing, has opened very interesting aspects in the non-linear dynamics of multimode devices. In particular, it exhibits an effective Kerr interaction, responsible for soliton generation under optical injection, as discussed in Ref. [12]. As shown in Ref. [13], the self-generation of solitons is shown to be very dependent on the existence of a small amount of optical backscattering. Finally, as discussed theoretically in Ref. [14], the fast gain recovery yields to the optical field a liquid behavior that enables comb generation through a quantum walk process in synthetic space.

Ongoing improvements in efficiency, miniaturization, and room-temperature operation have presently solidified QCLs’ role in both scientific research and commercial technologies. As global demand for sensitive, compact, and real-time spectroscopic tools continues to grow, QCLs are poised to remain at the forefront of photonic innovation.

Progress in quantum-engineered materials and heterogeneous integration with photonic circuits may open the door to fully integrated, smart sensing platforms – paving the way for the widespread deployment of QCL-based systems in both industrial and consumer technologies.

The evolution of QCL technology has driven remarkable progress across various areas of laser physics and practical applications, accompanied by a consistent rise in the number of research groups and companies engaged in the field globally, promising to inspire new avenues in laser physics.

## References

[1] Faist J., Capasso F., Sivco D. L., Sirtori C., Hutchinson A. L., Cho A. Y. (1994). Quantum cascade laser. *Science*.

[2] Kazarinov R. F., Suris R. A. (1971). Possibility of the amplification of electromagnetic waves in a semiconductor with a superlattice. *Sov. Phys. Semi-Cond.*.

[3] Szedlak R., Schwarz B., Strasser G. (2025). Surface-emitting ring quantum cascade lasers. *Nanophotonics*.

[4] Zhang K., Mayer R., Burghart D., Boehm G., Belkin M. A. (2025). Mid-infrared wavelength multiplexers on an InP platform. *Nanophotonics*.

[5] Marschick G. (2025). Micro-mirror aided mid-infrared plasmonic beam combiner monolithically integrated with quantum cascade lasers and detectors. *Nanophotonics*.

[6] Del Balzo L. (2025). Hybrid LiDAR–radar at 9 μm wavelength with unipolar quantum optoelectronic devices. *Nanophotonics*.

[7] Hu Q. (2025). From far-infrared detectors to THz QCLs. *Nanophotonics*.

[8] Gloor S. (2025). Compact, thermoelectrically cooled surface emitting THz QCLs operating in an HHL housing. *Nanophotonics*.

[9] De Vetter A. (2025). Extended spectral response of cavity-based terahertz photoconductive antennas and coherent detection of quantum cascade lasers. *Nanophotonics*.

[10] Liu H. (2025). High Q-contrast terahertz quantum cascade laser via bandgap-confined bound state in the continuum. *Nanophotonics*.

[11] Jirauschek C. (2025). Effective discrete-level density matrix model for unipolar quantum optoelectronic devices. *Nanophotonics*.

[12] Lugiato L., Prati F., Brambilla M., Luigi Columbo L. (2025). Solitons in ultrafast semiconductor lasers with saturable absorber. *Nanophotonics*.

[13] Seitner L., Schreiber M. A., Rinderle M., Pichel N., Haider M., Jirauschek C. (2025). Aspects of cavity engineering in THz quantum cascade laser frequency combs. *Nanophotonics*.

[14] Dikopoltsev A., Heckelmann I., Schneider B., Bertrand M., Faist J. (2025). The theory of the quantum walk comb laser. *Nanophotonics*.

